# Construction and characterization of the first *Arcobacter butzleri *- *Escherichia coli* shuttle vector

**DOI:** 10.1007/s00203-026-04874-x

**Published:** 2026-03-30

**Authors:** Adrián Salazar-Sánchez, Rodrigo Alonso, Aurora Fernández-Astorga, Ilargi Martínez-Ballesteros, Irati Martinez-Malaxetxebarria

**Affiliations:** 1 MikroIker Research Group, Department of Immunology, Microbiology, and Parasitology, Faculty of Pharmacy, University of the Basque Country EHU, Paseo de la Universidad 7, 01006 Vitoria-Gasteiz, Spain; 2 Bioaraba, Microbiology, Infectious Disease, Antimicrobial Agents, and Gene Therapy, 01006 Vitoria-Gasteiz, Spain

**Keywords:** *Arcobacter butzleri*, Gene function restoration, Genetic complementation, Shuttle vector

## Abstract

This study addresses a key limitation in the genetic characterisation of *Arcobacter butzleri*, an emerging food- and waterborne pathogen associated with gastrointestinal and systemic infections in humans and animals. Despite the identification of numerous putative virulence genes, functional validation has been hindered by the absence of suitable complementation tools. Here, we report the development and characterisation of the first shuttle vector capable of replication in both *Escherichia coli* and *A. butzleri*. A total of 117 *A. butzleri* isolates were screened for plasmids, leading to the identification and sequencing of a novel cryptic plasmid, pABRW13 (3,402 bp), from a waterborne strain. This plasmid revealed four open reading frames, including a putative replication-associated gene. These elements were incorporated into a novel shuttle vector, pIMM24 (7,425 bp), together with a tetracycline resistance cassette (*tetO*), using the pBluescript KS derivative pMW2 vector as backbone. The resulting construct successfully replicated in both hosts, exhibited stable maintenance in *E. coli* and moderate stability in *A. butzleri*, and conferred increased tetracycline resistance. Functional validation was achieved by complementation of a *fliS* knockout mutant, restoring motility to near wild-type levels. Overall, pIMM24 represents a significant advancement in the genetic toolkit available for *A. butzleri*, enabling gene complementation studies and facilitating deeper insights into its physiology and pathogenic mechanisms.

**Supplementary Information:**

The online version contains supplementary material available at 10.1007/s00203-026-04874-x.

## Introduction

*Arcobacter butzleri* is a Gram-negative bacterium widely distributed in nature that can be found in a broad diversity of niches, such as animals, environmental waters, and different types of food (for example, vegetables, meat, seafood, and dairy products) (Martinez-Malaxetxebarria et al. 2022). In fact, one of the main routes of transmission of this species to humans is through the consumption of contaminated and improperly treated food and/or water (Shange et al. 2019; Chieffi et al. 2020). In humans, the presence of this bacterium has been associated with various health issues including enteritis, severe diarrhea, bacteremia, septicemia, endocarditis, and peritonitis. Similarly, in animals, it has been linked to enteritis, stillbirths, and abortions (Chieffi et al. 2020).

To date, several virulence-associated genes have been described in *A. butzleri*, including those involved in adherence (*cadF*,* cj1349*,* hecA*,* hecB*), invasion and colonization (*ciaB*), motility (*flaA*,* flaB*,* flgH*,* motA*), and iron acquisition (*iroE*,* irgA*), as well as other putative factors such as *mviN*,* pldA*, and *tlyA* (Isidro et al. 2020; Chieffi et al. 2020). Comparative genomic analysis of 49 isolates further identified additional genes potentially contributing to pathogenicity, although their functional roles remain to be experimentally confirmed (Isidro et al. 2020). These include genes encoding a type IV secretion system (T4SS), invasion-related genes (*iamA*), regulators of iron uptake (*fur*), genes involved in capsular polysaccharide biosynthesis (*gmhA2*), chemotaxis genes (*cheA–cheY*), and the urease cluster *ureD(AB)CEFG*.

Targeted gene inactivation has enabled the association of several genes with specific functions in *A. butzleri*. For example, the flagellin gene *flaA* has been linked to motility (Ho et al. 2008); extracytoplasmic function sigma factors to the regulation of energy metabolism (Martinez-Malaxetxebarria et al. 2012); *flaAB*,* fliS*,* luxS*,* pta*, and *spoT* to adhesion through biofilm formation (Salazar-Sánchez et al. 2022); *ackA* and *lctP* to acetate and lactate metabolism, respectively (Szydlowski et al. 2020); and *cadF*,* ciaB*, and *flaAB* to adhesion and invasion of human Caco-2 and HT29-MTX cells (Baztarrika et al. 2024). These studies relied on genetic tools such as suicide vectors (e.g., pGEM-T Easy) or CRISPR-Cpf1 systems (Ho et al. 2008; Martinez-Malaxetxebarria et al. 2012; Szydlowski et al. 2020; Salazar-Sánchez et al. 2022). However, functional complementation of inactivated genes has not yet been achieved due to the lack of appropriate tools. As shown in Table 1, and in contrast to closely related bacteria such as *Campylobacter* spp. and *Helicobacter pylori*, no shuttle vectors are currently available for *A. butzleri*. The development of a shuttle vector capable of replication in two bacterial hosts is therefore essential for generating complemented mutants and advancing our understanding of this species.

**Table 1:** Different shuttle vectors available for genomic manipulation of bacteria phylogenetically close to *Arcobacter butzleri*

Shuttle vectors and bacterial hosts	References
*Campylobacter* spp. - *Escherichia coli*	
pIL550	(Labigne-Roussel et al. 1987)
pUOA13, pUOA15, pUOA17	(Wang and Taylor 1990a)
pUOA14, pUOA18, pYW69C	(Wang and Taylor 1990b)
pCHI15	(Waterman et al. 1993)
pRY107, pRY108, pRY111, pRY112	(Yao et al. 1993)
pMW10	(Wösten et al. 1998)
pGU0202	(Alfredson and Korolik 2003)
*Helicobacter pylori* - *Escherichia coli*	
pBHP489K	(Lee et al. 1997)
pHel2, pHel3	(Heuermann and Haas 1998)
pHP1	(Ando et al. 2000)
pSB13, pSB14, pSB17, pSB18	(Backert et al. 2005)
pDH37	(Croxen et al. 2007)
pTM117	(Carpenter et al. 2007)
pBHP69KH	(Joo et al. 2012)

Therefore, the aim of this study was to develop the first genetic tool to restore the function of previously inactivated genes in *A. butzleri*, capable of propagating and expressing in both, *Escherichia coli* and *A. butzleri*. This would facilitate more comprehensive studies of the species’ gene function and involvement in physiological processes, as well as the deciphering of hitherto unknown pathogenicity mechanisms.

## Materials and methods

### Bacterial strains, plasmids, and culture conditions

A total of 117 *A. butzleri* strains recovered from food products and water environments were tested for the presence of plasmids. All had been previously isolated from poultry (*n* = 14), raw milk (*n* = 12), mussels (*n* = 4), clams (*n* = 10), pork meats (*n* = 6), minced beef (*n* = 1), sewage (*n* = 54), and river waters (*n* = 16) during surveillance studies conducted in the North of Spain (Nieva-Echevarria et al. 2013; Alonso et al. 2018). In addition, the strain *A. butzleri* RM4018 (= CCUG 30485, ATCC 49616, LMG 10828, CECT 8221, DSM 8739) was used as wild-type (WT) in the different experiments performed, and the strains *Arcobacter cryaerophilus* CCUG 17,801 and *Arcobacter skirrowii* CCUG 10,374 were used in order to determine whether the function of replication identified on a cryptic plasmid of *A. butzleri* is also recognized by other species of the genus. The clinical isolate *Campylobacter jejuni* CH003, belonging to the MikroIker Research Group’s bacterial collection, was used to obtain a tetracycline resistance cassette. *E. coli* DH5α was used as host for cloning, propagation, and maintenance of the plasmids. Arcobacters were routinely grown in Brain Hearth Infusion (BHI) broth and on BHI agar (Oxoid, Basingstoke, UK) or Columbia blood agar plates (Oxoid, Basingstoke, UK), and incubated at 30 °C under aerobic conditions for 24–48 h. *E. coli* DH5α was routinely growth in Luria-Bertani (LB) broth (Condalab, Torrejón de Ardoz, Spain) or on LB agar (Condalab, Torrejón de Ardoz, Spain), under the same incubation conditions. *C. jejuni* CH003 was grown in the same media as *Arcobacter*, but at an incubation temperature of 42 °C in microaerobic conditions (GENbag microaer atmosphere generator; Biomérieux, Marcy-l’Étoile, France). When necessary, filtered-sterilized ampicillin (CAS: 69-52-3; Sigma-Aldrich, St. Louis, USA), kanamycin (CAS: 25389-94-0; NZYTech, Lisbon, Portugal), or tetracycline (CAS: 64-75-5; Sigma-Aldrich, St. Louis, USA) were added to the media at a final concentration of 100 µg/mL, 50 µg/mL, and 15 µg/mL, respectively. The plasmids used in this study are listed in Table 2.

**Table 2:** List of plasmids used in this study, their description and source

Plasmid	Features	Source or Reference
pGEM-T Easy	Cloning vector; Amp^r^; 3,015 bp	Promega^1^
pUC18	Cloning vector; Amp^r^; 2,686 bp	ThermoScientific^2^
pMW2	pBluescript KS M13 + :KmR (pILL550); Km^r^, Amp^r^; 4,452 bp	(Wösten 1997)
pABRW13	*A. butzleri* RW13 strain’s cryptic plasmid; 3,402 bp	This study
pGemOrfs	pGEM-T Easy with PCR obtained pABRW13 ORFs (1 to 4); Amp^r^; 5,172 bp	This study
pMW2Orfs	pMW2 with ORFs (1 to 4) from pABRW13; Km^r^; 6,601 bp	This study
pGemTET	pGEM-T Easy containing *tetO* gene with BamHI flanking restriction sites; Tet^r^; 5,337 bp	This study
pIMM24	*E. coli - A. butzleri* shuttle vector; Amp^r^, Tet^r^; 7,425 bp	This study
pIMM24_fliS	pIMM24 containing *fliS* gene with SacI flanking restriction sites; Amp^r^, Tet^r^; 9,181 bp	This study

^1^ Promega, Madison, USA. ^2^ ThermoScientific, Vilnius, Lithuania

### Preparation of competent cells and plasmid transformation

Chemically competent *E. coli* DH5α cells were prepared by the calcium chloride method using the solutions TBF1 [30 mM potassium acetate (CAS: 127-08-2), 100 mM potassium chloride (CAS: 7447-40-7), 10 mM calcium chloride (CAS: 10043-52-4), 50 mM manganese chloride tetrahydrate (CAS: 13446-34-9), and 15% glycerol (CAS: 56-81-5; Panreac, Barcelona, Spain)] and TBF2 [10 mM MOPS (CAS: 1132-61-2), 75 mM calcium chloride, 10 mM potassium chloride and 15% glycerol]. Briefly, 5 mL of LB broth were inoculated with a single colony of *E. coli* DH5α derived from an overnight culture grown at 37 °C on LB agar. After an overnight incubation at 37 °C with agitation, 1 mL of the resulting culture was transferred into 100 mL of fresh LB broth. Once the culture reached an optical density at 600 nm (OD_600_) between 0.4 and 0.5, corresponding to approximately three hours of incubation at 37 °C, it was divided into two 50 mL conical centrifuge tubes. Those were then incubated on ice for five minutes prior to undergoing a centrifugation process at 4,000 × g for 10 min at 4 °C (Gyrozen 1580R; Gyrozen, Gimpo, Korea). The supernatants were discarded, and the cell pellets were then gently resuspended in 40 mL of TBF1 solution. After a further five-minute incubation on ice, the samples were subjected to a second centrifugation under the same conditions, and the resulting pellets were resuspended in 4 mL of TBF2 solution. The suspensions were incubated on ice for 15 min prior to the process of aliquoting. The competent cells were subsequently stored at − 80 °C for a maximum period of four to six months. All reagents were obtained from Sigma-Aldrich, St. Louis, USA, unless otherwise specified.

Plasmids were introduced into competent *E. coli* DH5α cells by heat-shock transformation, and transformants were selected on LB agar supplemented with ampicillin, 80 µg/mL X-Gal (CAS: 7240-90-6; ThermoScientific, Vilnius, Lithuania), and 0.2 mM IPTG (CAS: 367-93-1; ThermoScientific, Vilnius, Lithuania). The heat-shock transformation procedure was conducted by mixing up to 1 µg of plasmid DNA with 50 µL of competent cells. The mixture was then subjected to an ice incubation period of between 20 and 30 min, after which a heat shock treatment at 42 °C was applied for 30 s. The cells were then rapidly returned to ice for 2 min. Subsequently, 1 mL of SOC medium was added to the suspension, and the cells were allowed to recover at 37 °C with gentle agitation for one hour.

Electrocompetent *Arcobacter* cells were prepared using an ice-cold sucrose/glycerol solution [272 mM sucrose (CAS: 57-50-1; NZYTech, Lisbon, Portugal) and 15% glycerol], as previously described (Martinez-Malaxetxebarria et al. 2012). Briefly, 5 mL of BHI broth were inoculated with a fresh single colony of *Arcobacter* spp. and incubated overnight at 37 °C with agitation. The 5 mL culture was diluted into 100 mL of fresh BHI broth. When the culture reached an OD_600_ of 0.2–0.6, corresponding to approximately 12–16 h of incubation at 30 °C, it was divided equally into two 50 mL conical centrifuge tubes and they were centrifugated at 3,000 × g for 60 min at 4 °C (Gyrozen 1580R). The supernatants were discarded, and the cell pellets were gently resuspended in 15 mL of ice-cold sucrose/glycerol solution and combined into a single tube. The samples were subjected to a second centrifugation under identical conditions. After discarding the supernatant, the washing procedure was repeated once more. The final pellet was resuspended in 0.5–1 mL of ice-cold sucrose/glycerol solution, aliquoted, and stored at -80 °C for a maximum period of four to six months. Plasmids were then introduced into the cells by electroporation. In brief, 1 µg of plasmid DNA was combined with 50 µL of competent *Arcobacter* cells within a 0.2 cm electroporation cuvette. An electric pulse was applied under conditions of 2.25 kV, 400 Ω, and 25 µF. The cells were then recovered in 1 mL of BHI medium, and left to incubate at room temperature for 10 min. Subsequently, the suspension was transferred to a 25 cm^2^ flask containing 2 mL of pre-warmed BHI, and the flask was then incubated at 30 °C with gentle agitation for a duration of three hours. The transformants were selected on Columbia blood agar supplemented with tetracycline.

### Cryptic plasmid DNA isolation and sequence analyses

Cryptic plasmid DNA was isolated from overnight liquid cultures using the GeneJET Plasmid Miniprep Kit (ThermoScientific, Vilnius, Lithuania). Plasmid sizes were determined by restriction digest with HindIII (ThermoScientific, Vilnius, Lithuania). All digestion products were electrophoresed in 2% agarose (Bio-Rad, Hercules, USA) gels in 1× TBE (Tris/Borate/EDTA) buffer with a 1:20,000 GelRed (Biotium, Fremont, USA) stock solution (10,000 x in water). Visualization was achieved using a UV transilluminator (ChemiDoc XRS system, Bio-Rad, Hercules, USA) with an analyst computer program (Quantity One XRD software, Bio-Rad, Hercules, USA).

Those plasmids that exhibited a single band and variable sizes after being digested with HindIII were selected for further analysis. The analysis consisted of gel extraction of the resulting band using the QIAEX^®^II Gel Extraction Kit (Qiagen, Hilden, Germany), cloning into the HindIII-cut pGEM-T Easy cloning vector (Promega, Madison, USA), heat-shock transformation into *E. coli* DH5α, and DNA sequencing by the primer-walking Sanger method. The latter was carried out at the General Services of the University of the Basque Country EHU (SGIker), initially using the universal sequencing primers M13F (CCCAGTCACGACGTTGTAAAACG) and M13R (AGCGGATAACAATTTCACACAGG). The sequences of the obtained recombinant plasmids were annotated and analyzed using the RAST (Rapid Annotations using Subsystems Technology) server (https://rast.nmpdr.org/) and the BLASTn (Basic Local Alignment Search Tool) alignment tool (https://blast.ncbi.nlm.nih.gov/Blast.cgi) under default settings.

### Amplification and cloning of the replication functions of plasmid pABRW13 and *tetO* gene of *C. jejuni* CH003

A 2,157 bp DNA sequence of the plasmid pABRW13 (3,402 bp, accession number NZ_MF136769.1) containing the replication functions was amplified by PCR using the primers RW13Orfs-F (A*ACATGT*TGTTCTGGTCTTAGAGTTAG) and RW13Orfs-R (A*ACATGT*AAATTATGAGTGGCATTGTG). These primers incorporate PciI restriction sites, which are shown in italics, and the Accuzyme DNA polymerase, a proofreading enzyme produced by Bioline (Memphis, USA). The amplified fragment was then cloned into the pGEM-T Easy cloning vector, and the resulting plasmid, designated pGemOrfs, was transformed into *E. coli* DH5α. White transformants were selected and grown overnight in LB broth supplemented with ampicillin at 37 °C. Thereafter, they were preserved in 25% glycerol at -80 °C.

To create a tetracycline resistance cassette, the *tetO* chromosomal gene from the tetracycline resistant *C. jejuni* CH003 strain was amplified by proofreading PCR using the TetOF and TetOR primers (Jeon et al. 2011), which had been modified by inserting a BamHI restriction site (*in italics*) and renamed TETO3-F (A*GGATCC*TTATTTTTGCATAAACAGATGATTAGTG) and TETO3-R (A*GGATCC*GCAAGCTG TTAAGCTAACTTGT). The amplified *tetO* gene (2,325 bp) was cloned into the pGEM-T Easy cloning vector and subsequently transformed into *E. coli* DH5α. The resistance of the transformants to tetracycline was tested by growing them on LB agar containing tetracycline. In order to preserve the resulting plasmid, pGemTET, the transformants were stored in 25% glycerol at -80 °C.

### pIMM24 plasmid construction and characterization

#### Construction

The pIMM24 *E. coli* – *A. butzleri* shuttle vector was constructed using the pMW2 plasmid as the backbone (Wösten  1997), with its original kanamycin resistance cassette replaced by a tetracycline resistance one and the replication functions of *A. butzleri* inserted. The procedure is outlined in Figure S1 (Online Resource). All the enzyme digestions were carried out using enzymes purchased from ThermoScientific (ThermoScientific, Vilnius, Lithuania), with the option of FastDigest enzymes when those were available. Linearized vectors were then subjected to a dephosphorylation process using FastAP Thermosensitive Alkaline Phosphatase (ThermoScientific, Vilnius, Lithuania) to prevent recircularization. T4 DNA ligase (ThermoScientific, Vilnius, Lithuania) was used for both ligations and self-ligations. All DNA extractions from the gel were performed using the QIAEX II Gel Extraction Kit, always starting from a 1% agarose gel in Tris-Acetate-EDTA (TAE) buffer (ThermoScientific, Vilnius, Lithuania). Briefly, the pMW2 plasmid was linearized by restriction enzyme digestion with PciI and then ligated to the 2,157 bp fragment containing the functions of replication of pABRW13, which was obtained from the PciI-digested pGemOrfs. The pMW2Orfs plasmid (5,172 bp) was thus obtained. The final pIMM24 plasmid was created by removing the kanamycin resistance cassette from pMW2Orfs through BamHI digestion, and then ligating the linearized pMW2Orfs with the tetracycline resistance cassette, which was obtained from the BamHI digested pGemTET. The pIMM24 *E. coli* – *A. butzleri* shuttle vector was transformed into *E. coli* DH5α, and transformants were selected based on their resistance to tetracycline. The cells were preserved in glycerol, as above indicated.

#### Sequence determination

The sequencing of pIMM24 was performed using both, Nanopore and Illumina technologies. The Nanopore sequencing was carried out with the Rapid Sequencing Kit and a MinION sequencer (Oxford Nanopore Technologies, OX4 4DQ, United Kingdom). The Illumina sequencing was performed at Eurofins (Eurofins, Ebersberg, Germany) using a NovaSeq 6000 sequencer. The shuttle vector sequence was obtained by merging all data from both sequencing processes. The Flye assembler (https://github.com/fenderglass/Flye) (Kolmogorov et al. 2019) and the OnRamp bioinformatics tool (https://onramp.boylelab.org/) (Mumm et al. 2023) were utilised in this process.

#### Transformation efficiency determination

The transformation efficiency of the plasmid was examined in *E. coli*,* A. butzleri*, *A. cryaerophilus*, and *A. skirrowii*. For this purpose, 1 µg of the constructed shuttle vector was transformed into *E. coli* DH5α by heat-shock, or into *Arcobacter* spp. by electroporation. The transformed cells were then plated on blood agar plates supplemented with tetracycline and incubated at 30 °C for a period of between two to five days. After the incubation, the colony forming units (CFUs) were counted, and a maximum of 15 CFUs per transformant were transferred into three millilitres of tetracycline supplemented BHI. The samples were then incubated at 30 °C for 24–48 h with agitation. The transformation efficiency was determined by calculating the number of transformed CFU per µg of shuttle vector DNA (CFU/µg). In order to confirm the successful transformation of the shuttle plasmid, genomic DNA and transformed plasmid DNA were extracted from a minimum of six transformants. The boiling method and GeneJET Plasmid Miniprep Kit were used, respectively. Subsequently, the extracted DNA was used for PCR amplification of the *tetO* gene with the TETO3-F and TETO3-R primers. The assay was performed in triplicate.

#### Stability determination

The stability of the plasmid was analysed using a protocol based on those proposed by Nishida et al. and Kang et al. (Nishida et al. 2017; Kang et al. 2020). *E. coli* DH5α and *A. butzleri* RM4018 were transformed with pIMM24 and subsequently plated on BHI agar supplemented with tetracycline. A fresh single colony of each transformant was used to inoculate 5 mL of tetracycline supplemented BHI broth, which was then incubated at 30 °C for 24 h under shaking conditions. The broth was subsequently diluted to 1% in 5 mL of fresh BHI broth, and the incubation was continued under the same conditions. This process was repeated on a daily basis for a period of a week. The number of viable cells in each suspension (CFU/mL) was determined by the plate count method on both, BHI agar and BHI agar with tetracycline, at 24 h intervals. The stability of the plasmid was calculated by dividing the CFU counts on BHI agar with tetracycline by the CFU counts on BHI agar and multiplying by 100 to obtain a percentage. The experiment was conducted in triplicate.

#### Plasmid-conferred tetracycline resistance determination

To determine the tetracycline resistance conferred by the shuttle plasmid, the susceptibility of WT and pIMM24-transformed *E. coli* DH5α and *A. butzleri* RM4018 strains to tetracycline was tested. The minimal inhibitory concentration (MIC) for tetracycline of the WT and transformant strains was determined by the gradient strip diffusion method (MIC Test Strip; Liofilchem, Roseto degli Abruzzi, Italy). The specifications of the European Committee on Antimicrobial Susceptibility Testing (EUCAST) were followed for *E. coli*. However, in the absence of specific recommendations, the standard inoculation procedure was adapted for *Arcobacter* strains, which were seeded on the surface of a blood agar plate by pouring 900 µL of an overnight liquid culture in BHI. The strains were classified as either susceptible or resistant according to the breakpoints proposed by the EUCAST (Version 15.0) (EUCAST 2025) for *Enterobacterales* in the case of *E. coli* (where the breakpoint is defined as Susceptible < 0.5 mg/L < Resistant), and for *C. jejuni/coli* in the case of *A. butzleri* (where the breakpoint is defined as Susceptible < 2.0 mg/L < Resistant).

#### Plasmid copy number determination

Two PCR techniques were initially applied: a qPCR using the SYBR Premix Ex Taq (Perfect Real Time) master mix (Takara Bio, Sweden) and the AppliedBiosystems StepOne Real-Time PCR System instrument (ThermoScientific, Vilnius, Lithuania), and a ddPCR using the QX200 ddPCR EvaGreen Supermix (Bio-Rad, Hercules, USA) on a ddPCR system with 96-well PCR plates (Bio-Rad, Hercules, USA). The latter was externalized to the Severo Ochoa Molecular Biology Centre (CSIC, Madrid, Spain). The genes selected as targets were *aroL* for *E. coli*, *cysD* for *A. butzleri*, and *tetO* for the pIMM24 plasmid. In parallel, a complementary estimation was performed, starting by specifically isolating plasmid DNA using the GeneJET plasmid extraction kit (ThermoScientific, Vilnius, Lithuania). These extractions were carried out from the strains *A. butzleri* RM4018, *A. butzleri* RM4018 + pIMM24, *E. coli* DH5α, and *E. coli* DH5α + pIMM24. An aliquot of the extracted DNA (5 µL) was subjected to agarose gel electrophoresis (0.8% agarose, 100 V, 90 min) to visualise the plasmid DNA. The copy number was calculated using the following equation, in which *C*_*DNA*_ represents the DNA concentration quantified using a NanoDrop 2000 (ThermoScientific, Vilnius, Lithuania), *N*_*A*_ corresponds to Avogadro’s number (6.022 × 10^23^), *l* denotes the length of the vector (7,425 bp), *ng* is the conversion factor to nanograms (10^9^), and *ω*_*bp*_ represents the average molecular weight of a base pair (660 Da for double-stranded DNA):

$$\:DNA\:copies/\mu\:L\:=\:\frac{{C}_{DNA}\times\:{N}_{A}}{l\:\times\:ng\:\times\:\:{\omega\:}_{bp}}$$

### Analysis of the effect of the pIMM24 plasmid on bacterial growth

Growth analyses were conducted to determine whether the acquisition of pIMM24 could influence in the growth of the transformed cells. For this purpose, the growth curve profiles of WT and pIMM24-transformed *E. coli* DH5α and *A. butzleri* RM4018 strains were obtained in BHI or BHI supplemented with tetracycline, following a procedure that has been used in a previous work (Salazar-Sánchez et al. 2022). The optical density (OD_550_) was monitored at various time points during a 24 h period using a Synergy™ HT plate reader (BioTek Instruments Inc., USA). The exponential growth rates, expressed as generations per hour ± standard error, were calculated from three independent experiments.

### Functional validation of the pIMM24 shuttle vector

The utility of the constructed shuttle vector was verified by using it to complement *A. butzleri* CZ6Δ*fliS*, a previously constructed knockout (KO) mutant strain for the *fliS* gene (Salazar-Sánchez et al. 2022). Briefly, the complete *fliS* gene of *A. butzleri* was subjected to a proofreading PCR, using the SacI endonuclease restriction site (*in italics*) containing primers cfliS-F_SacI (*GAGCTC*ACGGATTCTTCTTTGG) and cfliS-R_SacI (*GAGCTC*AAAGGTGCA GGTTTAG) and the DNA of *A. butzleri* RM4018 as template. The resulting amplicon (1,762 bp) was ligated with the SacI-linearized pIMM24 to create the plasmid vector pIMM24_fliS (9,181 bp), which was transferred into electrocompetent *A. butzleri* CZ6Δ*fliS* cells by electroporation. The acquisition of the pIMM24_fliS by those transformants selected on tetracycline containing blood agar plates was confirmed by PCR using the cfliS-F_SacI and cfliS-R_SacI primer-pair. In order to ascertain whether the function that has been lost in the KO strain had been restored, the motility of the WT, KO mutant, and complemented *A. butzleri* CZ6 strains was tested on semisolid thioglycolate agar plates (thioglycolate medium containing 0.4% bacteriological agar) (Scharlau, Sentmenat, Spain) according to Salazar-Sánchez et al. (Salazar-Sánchez et al. 2022). The motility assay was performed in triplicate on three independent occasions.

## Statistical analyses

IBM SPSS Statistics 26 software (IBM Corp., New York, USA) was used for statistical analyses. Normalities were assessed by the Shapiro-Wilk test, and comparison between groups was conducted using the non-parametrical Kluskal-Wallis test. Significant differences were established at p values of < 0.05.

## Results

### Cryptic plasmid DNA isolation and sequence analyses

In total, 11 out of the 117 (9.4%) *A. butzleri* strains that were analysed were found to carry plasmids. The plasmids were detected in 54.5% (*n* = 6) of the strains isolated from raw milk, 27.3% (*n* = 3) from river waters, 9.1% (*n* = 1) from pork meats, and 9.1% (*n* = 1) from sewage. The strains AB-L7, AB-RW3, and AB-RW13, isolated the first from milk, and the other two from river waters, yielded a single plasmid band and their plasmids were selected for HindIII digestion. After the digestion, the pABRW13 plasmid resulted in a single band of approximately 3,400 bp, which was cloned into pGEM-T Easy and Sanger sequenced.

**Fig. 1:**
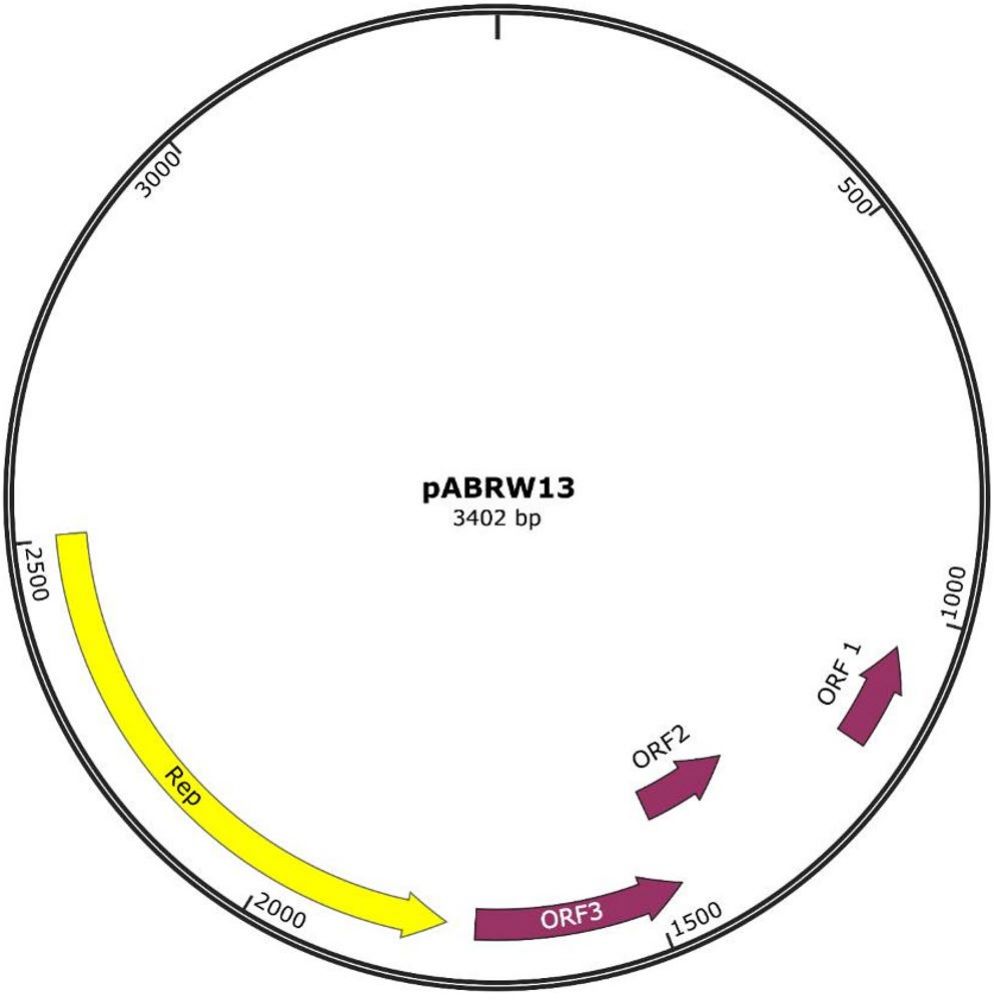
Genetic map of the pABRW13 cryptic plasmid (accession number MF136769.1). The length and direction of the four identified ORFs are indicated by arrows. Different restriction sites are shown too

The plasmid pABRW13, which has a total length of 3,402 bp and a GC content of 28%, is shown in Fig. 1. The RAST server identified four open reading frames (ORFs) in it. Based on BLASTn alignment, three were found to be devoiced of any known function, and one (ORF4, 741 bp) was associated with replication functions in *Campylobacter coli* and *A. butzleri*. Specifically, with more than a 65% of query coverage, ORF4 shared 67% sequence identity with a replication initiation protein in *C. coli* (OQ553938.1, CP068585.1, CP058338.1, NC_007143.1, and CP007185.1) and 65% with another in *A. butzleri* (NC_025125.1). The pABRW13 plasmid sequence was deposited at GenBank under the accession NZ_MF136769. The version described in this paper is version NZ_MF136769.1.

### Cloning of replication functions and tetracycline resistance cassette

In order to ensure that the final shuttle plasmid contained the required replication functions of *A. butzleri*, and considering that none of the four identified pABRW13 ORFs was particularly large, all four ORFs (2,143 bp) were amplified and cloned into the pGEM-T Easy vector, resulting in the successful creation of the pGemOrfs plasmid. In parallel, the *tetO* gene (2,281 bp) of *C. jejuni* CH003 was successfully cloned into the same cloning vector. It was demonstrated that *E. coli* DH5α transformants possessing pGemTET were able to grow on LB medium supplemented with tetracycline, thus verifying the expression of the tetracycline resistance cassette.

### pIMM24 plasmid construction and characterization

**Fig. 2:**
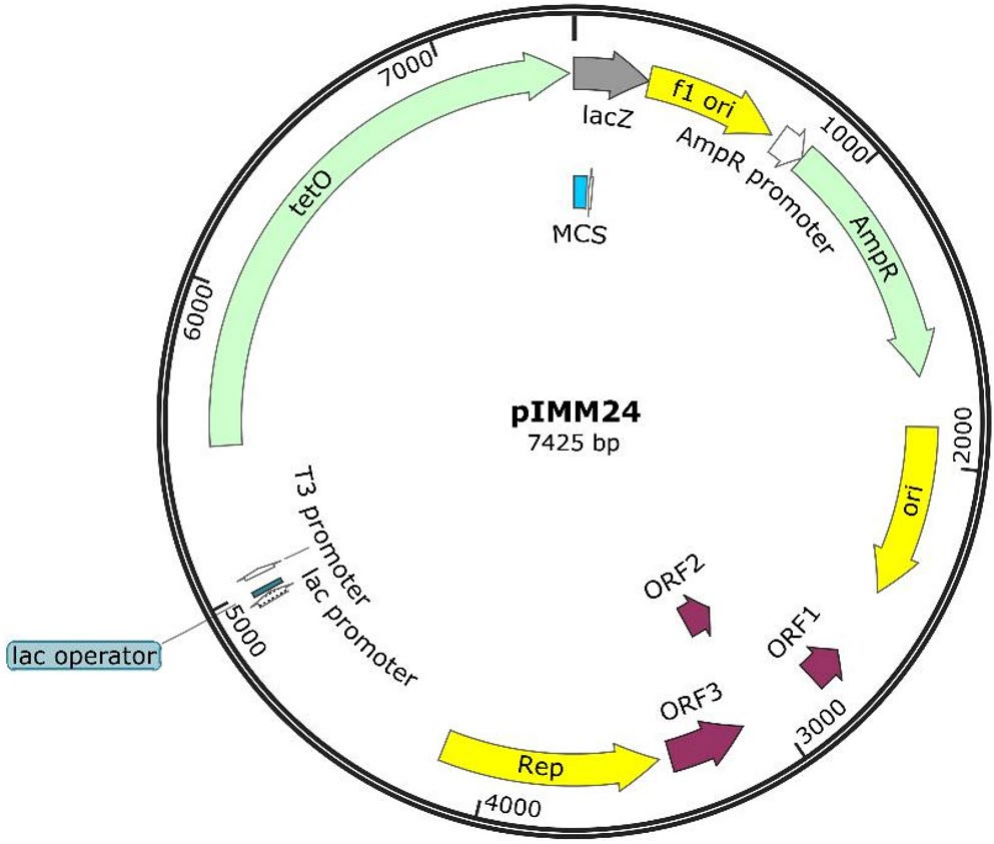
Genetic map of the pIMM24 *Escherichia coli* – *Arcobacter butzleri* shuttle vector (accession number PP129559.1). The arrows indicate the length and direction of the different genes and ORFs

The construction of the final shuttle plasmid was successfully achieved, with the pMW2 plasmid (Wösten 1997) serving as the base. The pIMM24 plasmid is 7,425 bp in length and has a GC content of 40.2%, as illustrated in Fig. 2. It contains regions that are essential for the replication of *A. butzleri* and *E. coli*, in addition to the tetracycline resistance gene *tetO* and the ampicillin resistance cassette. Furthermore, it includes a multi-cloning site after the *tetO* gene, with single restriction sites for the Bsu15I, ClaI, KpnI, SmaI, and XhoI endonucleases. The pIMM24 sequence data has been deposited at GenBank under the accession number PP129559. The version described in this paper is version PP129559.1.

**Fig. 3:**
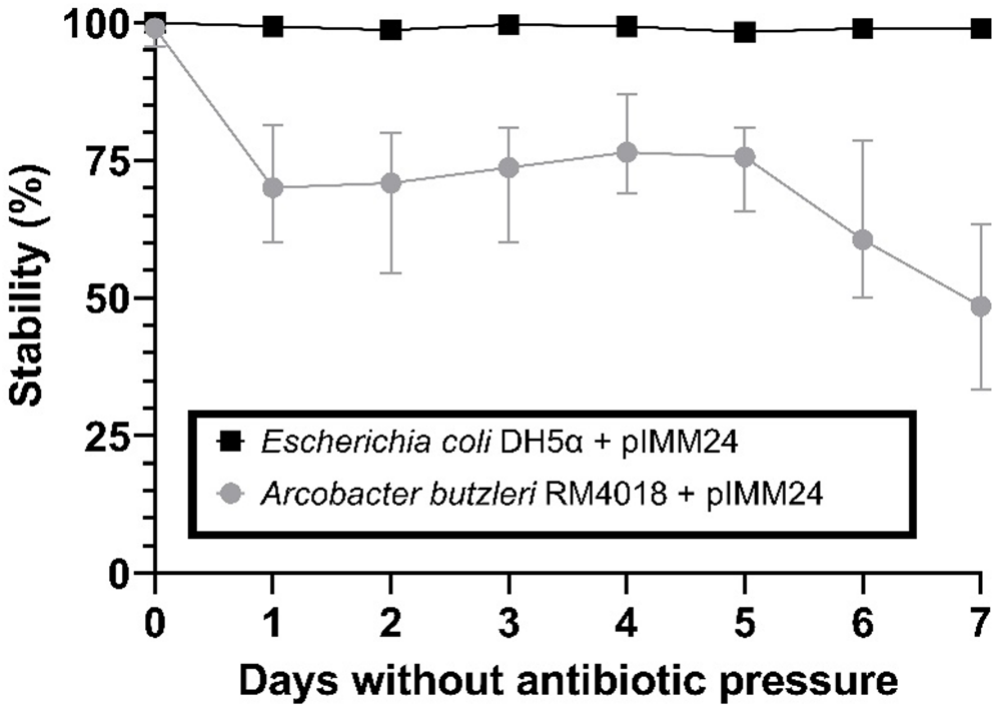
Results of the stability assays obtained from the pIMM24-transformed *Escherichia coli* DH5α and *Arcobacter butzleri* RM4018 strains

Based on performed assays, pIMM24 is capable of replication in *E. coli* and *A. butzleri*, yet this ability is absent in *A. cryaerophilus* and *A. skirrowii*. The plasmid transformation efficiency by both, heat-shock and electroporation, was found to be 4,572 ± 2,351 CFU/µg of vector in *E. coli* DH5α and of 32 ± 7 CFU/µg of vector in *A. butzleri* RM4018. The stability assays demonstrated that the shuttle vector is highly stable in *E. coli* DH5α, maintaining 100% stability for a minimum of seven days in a growth media devoid of antibiotic pressure (BHI). However, in the case of *A. butzleri*, the shuttle vector demonstrated reduced stability, maintaining a percentage of approximately 65–75% from the initial measurement on day one to day five. Thereafter, a decline was observed until it attained a stability level of 50% in the absence of antibiotic pressure, as shown in Fig. 3. The raw data from the stability assay can be found in Table S1.

Regarding the tetracycline resistance that is conferred to the host cells by the plasmid pIMM24, the *A. butzleri* and *E. coli* transformant strains exhibited a MIC for tetracycline of 32 and 24 µg/mL, respectively, while the parental strains displayed a MIC of 8 and 0.25 µg/mL. Consequently, the acquisition of the pIMM24 plasmid resulted in a 4-fold and 96-fold increase in the natural MIC of the WT *A. butzleri* and *E. coli* strains, respectively.

The precise vector copy number in *E. coli* and *A. butzleri* could not be determined using qPCR or ddPCR. Nevertheless, agarose gel electrophoresis confirmed the presence of the pIMM24 vector in the strains harbouring it (see Figure S2), and the estimation assay indicated approximately 1.26 × 10^10^ plasmid copies in *E. coli* DH5α + pIMM24 and 2.62 × 10^8^ copies in *A. butzleri* RM4018 + pIMM24. These results suggest that the vector is present at an approximate 1:48 ratio in *A. butzleri* relative to *E. coli*. Individual DNA quantifications for each strain are presented in Table S2.

### Bacterial growth analysis

Growth rates of 0.291 ± 0.007 and 0.251 ± 0.005 generations per hour were obtained for the WT and pIMM24-transformed strains of *E. coli* DH5α, respectively. The WT strain of *A. butzleri* RM4018 showed a growth rate of 0.201 ± 0.277 generations per hour, while the transformant strain demonstrated a growth rate of 0.146 ± 0.016 generations per hour. The results of the statistical analyses indicated that there was no significant difference between the growth rates of the WTs and the pIMM24-transformed *E. coli* DH5α (*p* = 0.583) and *A. butzleri* RM4018 (*p* = 0.139) strains. It is evident from the results obtained that the acquisition of the pIMM24 plasmid does not exert any influence on the growth of the transformants.

### Functionality of pIMM24

The successful electrotransformation of the plasmid pIMM24_fliS into the strain *A. butzleri* CZ6Δ*fliS* was indicated by the appearance of colonies on blood agar plates supplemented with tetracycline. The use of PCR, utilising the primers cfliS-F_SacI and cfliS-R_SacI, confirmed the presence of CZ6Δ*fliS* transformant strains carrying pIMM24_fliS (*A. butzleri* CZ6 complemented mutant strains), since two bands were obtained for all of them. Specifically, the PCR product exhibited a 3,129 bp band, which was indicative of the chromosomal KO *fliS* gene, along with an additional 1,762 bp band corresponding to the plasmidic intact gene that had been introduced by the pIMM24_fliS complementation vector.

**Fig. 4:**
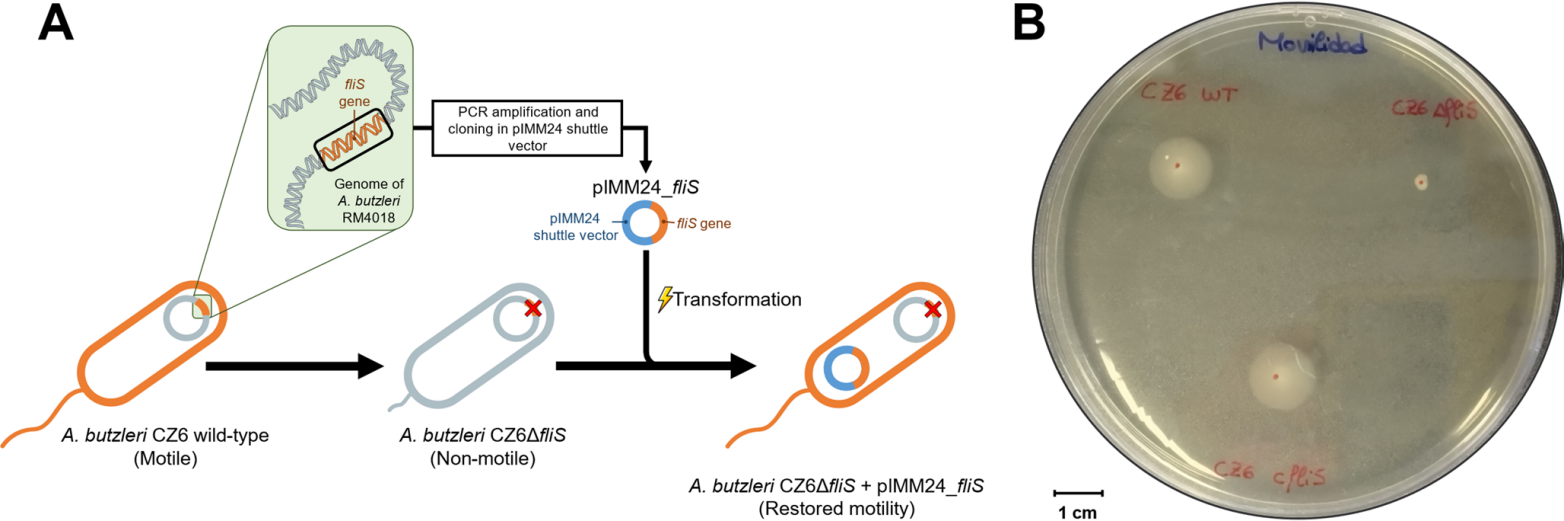
Functional validation process of the pIMM24 shuttle vector: (A) schematic representation of the assay carried out and (B) picture of a representative motility assay of the wild-type (CZ6 WT), *fliS*-knockout (CZ6 Δ*fliS*) and *fliS*-knockout complemented with the pIMM24_fliS vector (CZ6 c*fliS*) CZ6 strains

As demonstrated in Fig. 4, the motility assays conducted with the WT *A. butzleri* CZ6 strain along with its derivative strains, the CZ6Δ*fliS* KO mutant and the pIMM24_fliS complemented CZ6Δ*fliS* mutant, sustained the functionality of the constructed shuttle plasmid. The WT CZ6 exhibited a motility halo diameter of 14.17 ± 1.33 mm, the CZ6Δ*fliS* mutant one of 2.44 ± 0.73 mm, and the complemented strain one of 10.00 ± 3.29 mm. The differences between the WT and the KO mutant strains, and between the CZ6Δ*fliS* and complemented mutant, were both significant (*p* = 0.000 and *p* = 0.021, respectively). However, the difference between the WT and the complemented mutant was not significant (*p* = 0.182). This findings suggest that the motility in the CZ6Δ*fliS* mutant was restored by acquiring and maintaining the pIMM24_fliS plasmid.

## Discussion

Since the genus *Arcobacter* was first described in 1991, the number of studies on it has steadily increased, particularly on *A. butzleri*, the species that has attracted the most interest. However, only a limited number of studies have reported the presence of plasmids in the genus *Arcobacter* (Harrass et al. 1998; Douidah et al. 2014; On et al. 2019; Müller et al. 2020; Zhou et al. 2022). Currently, 28 *Arcobacter* plasmid sequences are available for consultation at the National Center for Biotechnology Innovation (NCBI), 11 of which are from *A. butzleri* and were mainly isolated from human (*n* = 3) and poultry (*n* = 7) sources. Therefore, the isolation of the new cryptic plasmid pABRW13, the first to be isolated from a waterborne *A. butzleri* strain, expands the available database of native *A. butzleri* plasmids. The presence of plasmids in *A. butzleri* strains does not appear to be a common, as suggested by the results of this study, as well as by those of Douidah et al. (Douidah et al. 2014), and Harrass et al. (Harrass et al. 1998) who detected plasmids, respectively, in only 7.3% and 23.6% of the 123 and 89 *A. butzleri* strains examined. Similarly, data from previous studies publicly available at NCBI, and the results presented here indicate that the plasmids extracted from *A. butzleri* are generally small (between 2,000 and 6,000 bp), although four larger plasmids (more than 25,000 bp) have also been reported (Harrass et al. 1998; Douidah et al. 2014).

An important step in plasmid vector design is selecting an antibiotic resistance cassette to enable the selection of the transformants of interest. Common antibiotics used for selecting genetically modified bacteria include ampicillin, kanamycin, tetracycline, erythromycin, and chloramphenicol. However, many *A. butzleri* strains display intrinsic resistance to ampicillin, with reported prevalence ranging from 50 to 90% (Shah et al. 2013; Rahimi 2014; Shirzad Aski et al. 2016; Šilha et al. 2017; Rathlavath et al. 2017; Vicente-Martins et al. 2018; Jehanne et al. 2022), making ampicillin resistance genes unsuitable as selection markers in this species. *A. butzleri* strains are generally susceptible to kanamycin (Rahimi 2014; Shirzad Aski et al. 2016; Rathlavath et al. 2017), making kanamycin resistance genes suitable markers for genetically modified arcobacters. Indeed, kanamycin resistance cassettes have been successfully employed in gene inactivation studies of *Arcobacter* spp. However, construction of a complementation vector requires a different marker for transformant selection. Although chloramphenicol resistance has previously been used for genetic modification of arcobacters (Ho et al. 2008), it was not considered here due to the high variability in chloramphenicol resistance among *A. butzleri* strains, with reported prevalence ranging from less than 10% (Shah et al. 2013; Rahimi 2014; Rathlavath et al. 2017) to more than 70% (Vicente-Martins et al. 2018; Khodamoradi and Abiri 2020; Khan et al. 2024). Tetracycline resistance has also been reported to be highly variable in *A. buztleri*, ranging from below 10% (Rahimi 2014; Shirzad Aski et al. 2016; Šilha et al. 2017; Rathlavath et al. 2017; Khodamoradi and Abiri 2020; Khan et al. 2024) to above 80% (Vicente-Martins et al. 2018; Lameei et al. 2022; Jehanne et al. 2022). Nevertheless, a 2019 meta-analysis on antibiotic resistance in *A. butzleri* (Ferreira et al. 2019) estimated overall resistance levels to be very low, below 6%. Tetracycline resistance in *A. butzleri* is typically mediated by genes such as *tetA*, *tetW*, and *tetO* (Sciortino et al. 2021; Barel and Yildirim 2023). Although the prevalence of *tetO* appears to be nearly zero (Chaiyasaen et al. 2023), it has been associated with high MIC values and a resistant phenotype (Lameei et al. 2022; Jehanne et al. 2022). For those reasons, *tetO* was chosen as the selectable marker in the present study. In line with previous reports, transformants harbouring pIMM24 showed a 4- to 96-fold increase in the MIC of tetracycline. Notably, studies demonstrating high tetracycline resistance in *Arcobacter* often apply the *C. jejuni/coli* EUCAST breakpoint of 2 µg/mL (EUCAST 2025), which is notably lower than the concentration typically used for the selection of tetracycline-resistant mutants (10–15 µg/mL). This discrepancy is illustrated by Jehanne et al. (Jehanne et al. 2022): using the 2 µg/mL breakpoint, 82% of the 101 strains analysed were classified as resistant, whereas applying a higher cut-off value (8 µg/mL), resistance reduced to 1%. Similarly, Vicente-Martins et al. (Vicente-Martins et al. 2018) reported MIC_50_ and MIC_90_ values of 8 µg/mL and 16 µg/mL, respectively, for 65 *A. butzleri* strains. Together, these finding indicate that using *tetO* as a selectable marker and selecting mutants at tetracycline concentrations above 8 µg/mL is a suitable strategy for the genetic manipulation of *A. butzleri*.

To be effective, complementation vectors, those designed to complement or restore the function of a chromosomal gene, must contain both replication functions compatible with the host and a selection marker. In this study, cloning the four ORFs of the cryptic plasmid pABRW13 into the pMW2 plasmid enabled successful replication of the resulting shuttle vector pIMM24 in *A. butzleri*. In contrast, no transformants of *A. cryaerophilus* or *A. skirrowii* were obtained (transformation efficiency of 0 CFU/µg pIMM24), indicating that, under the condition tested, the replication origin derived from *A. butzleri* is not functional in these non-*A. butzleri Arcobacter* species. Although additional species should be evaluated to better define the host range of the vector, similar species-specific replication constraints for shuttle vectors within the same genus have been reported previously (Song et al. 2018; Xie et al. 2025), supporting our observations. Ideally, a complementation vector should replicate and be stably maintained across multiple species of the genus. In this context, identifying a origin of replication conserved among *Arcobacter* species would be highly valuable and represents a key objective for future studies.

The vector constructed in this study showed lower transformation efficiency than vectors previously reported in the literature (Wang and Taylor 1990a). This may be related to the transformation methods used (heat shock in *E. coli* and electroporation in *A. butzleri*), which may not be optimal for plasmid introduction in these hosts. Alternatively, the reduced efficiency may reflect intrinsic properties of the plasmid or experimental parameters such as plasmid quantity and electroporation voltage (Wang and Taylor 1990a; Alfredson and Korolik 2003). The variability observed between experiments further indicates that optimisation of the transformation protocol is required to improve efficiency and reproducibility.

The vector also exhibited moderate stability in *A. butzleri*. Although lower than that observed in *E. coli*, this stability appears sufficient for complementation assays under antibiotic selection. This behaviour may be associated with the replication region derived from the cryptic plasmid pABRW13, which contains the replication functions of *A. butzleri*. The origin of replication, together with plasmid copy number, has been shown to play an important role in plasmid stability (Standley et al. 2019; Zhao et al. 2020). Overall, these results highlight the need to identify improved origins of replication to develop more stable and versatile shuttle vectors. Nevertheless, the vector described in the present study represents a significant advance for genetic studies in *A. butzleri*.

In conclusion, this work has made it possible to construct the first shuttle vector capable of replicating and expressing itself in both *E. coli* and *A. butzleri*. This valuable tool for the genetic modification of *A. butzleri* enables genetic complementation of this species, which is of great interest in terms of characterising genes.

## Supplementary Information

Below is the link to the electronic supplementary material.

Supplementary Material 1

Supplementary Material 2

## Data Availability

The data underlying this article are available in the article, in its online supplementary material, and in the GenBank Nucleotide Database at https://www.ncbi.nlm.nih.gov/nucleotide/. The latter can be accessed with NZ\_MF136769.
