# Supplementary material for: Construction and characterization of the first Arcobacter butzleri - Escherichia coli shuttle vector
Source: Arch Microbiol. 2026 Mar 30;208(6):295. doi: 10.1007/s00203-026-04874-x (PMC13035637; doi:10.1007/s00203-026-04874-x)
Supplement: Supplementary file 2 — Supplementary Material 2 [file 203_2026_4874_MOESM2_ESM.docx]

**Construction and characterization of the first *Arcobacter butzleri – Escherichia coli* shuttle vector**

Adrián Salazar-Sánchez^1,2^, Rodrigo Alonso^1,2^, Aurora Fernández-Astorga^1^, Ilargi Martínez-Ballesteros^1,2^ and Irati Martinez-Malaxetxebarria^1,2,^*

^1^ MikroIker Research Group, Department of Immunology, Microbiology, and Parasitology, Faculty of Pharmacy, University of the Basque Country EHU, Paseo de la Universidad 7, 01006 Vitoria-Gasteiz, Spain.

^2^ Bioaraba, Microbiology, Infectious Disease, Antimicrobial Agents, and Gene Therapy, 01006 Vitoria-Gasteiz, Spain.

* Corresponding author: Irati Martinez-Malaxetxebarria; Department of Immunology, Microbiology, and Parasitology, Faculty of Pharmacy, University of the Basque Country EHU, Paseo de la Universidad 7, 01006 Vitoria-Gasteiz, Spain; Email: [irati.martinez@ehu.eus](mailto:irati.martinez@ehu.eus); Phone number: +34 945 01 3471.

ORCID of the authors, respectively: 0000-0002-2454-4753, 0000-0002-8129-4850, 0000-0003-0509-3093, 0000-0002-8867-1487, and 0000-0002-6576-6707.

**SUPPLEMENTARY MATERIAL**


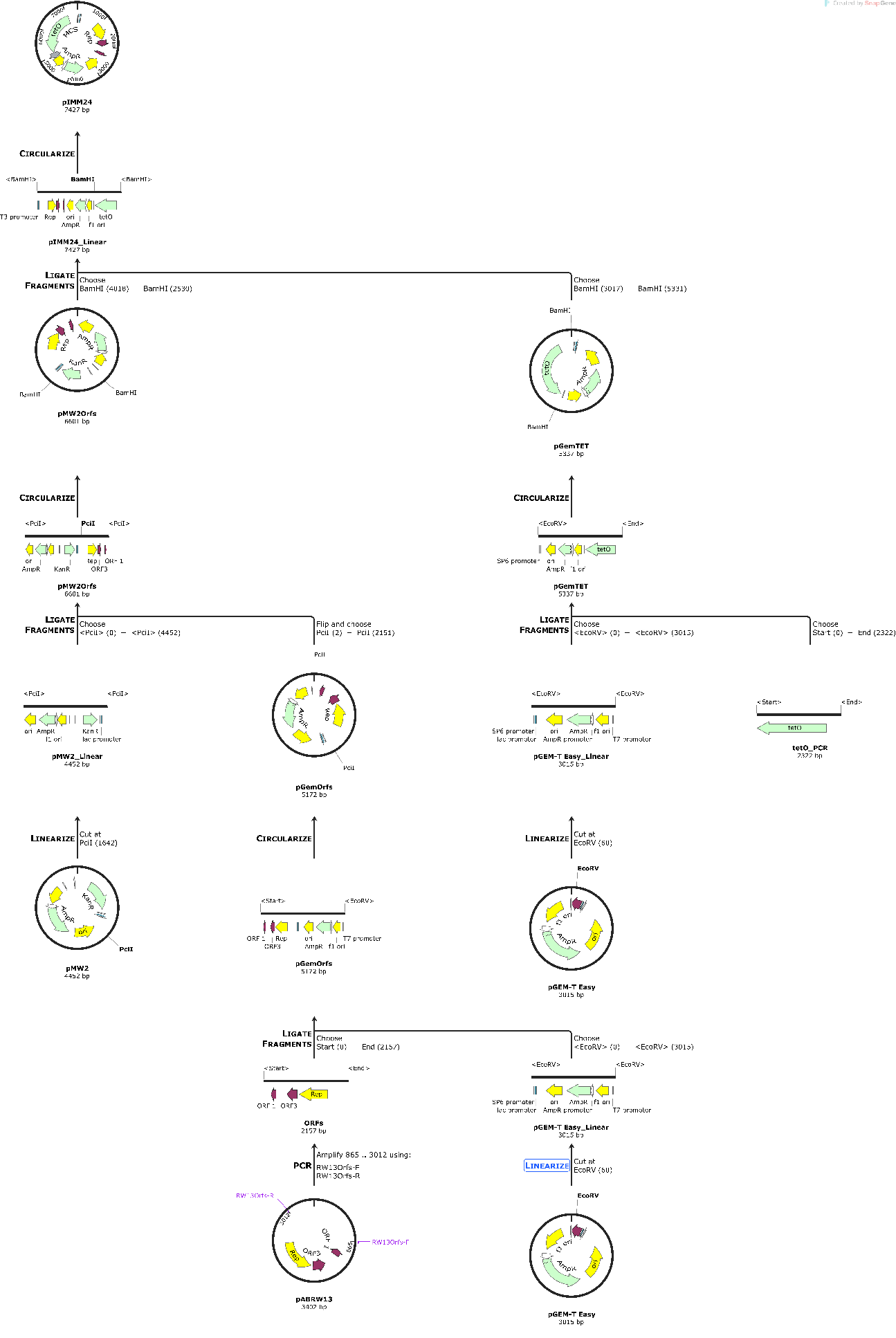


**Figure S1.** Schematic diagram of the construction of the pIMM24 vector.

**Table S1.** Raw data from the stability assay.

| **Strain** | **Day** | **BHI_UFC/mL** | **BHI+Tetra_UFC/mL** | **Stability** |
| --- | --- | --- | --- | --- |
| RM4018+pSh-1 | 0 | 9.67E+08 | 9.67E+08 | 100.00% |
| RM4018+pSh-1 | 1 | 1.80E+09 | 1.47E+09 | 81.48% |
| RM4018+pSh-1 | 2 | 7.33E+09 | 5.87E+09 | 80.00% |
| RM4018+pSh-1 | 3 | 5.67E+09 | 3.40E+09 | 60.00% |
| RM4018+pSh-1 | 4 | 1.03E+09 | 7.13E+08 | 69.00% |
| RM4018+pSh-1 | 5 | 3.07E+09 | 2.45E+09 | 80.00% |
| RM4018+pSh-1 | 6 | 3.00E+09 | 1.50E+09 | 50.00% |
| RM4018+pSh-1 | 7 | 1.30E+09 | 6.33E+08 | 48.72% |
| RM4018+pSh-2 | 0 | 2.53E+09 | 2.57E+09 | 101.32% |
| RM4018+pSh-2 | 1 | 2.00E+09 | 1.20E+09 | 60.00% |
| RM4018+pSh-2 | 2 | 6.33E+09 | 4.94E+09 | 78.00% |
| RM4018+pSh-2 | 3 | 6.33E+09 | 5.07E+09 | 80.00% |
| RM4018+pSh-2 | 4 | 5.00E+09 | 3.67E+09 | 73.33% |
| RM4018+pSh-2 | 5 | 2.53E+09 | 1.67E+09 | 65.79% |
| RM4018+pSh-2 | 6 | 3.33E+09 | 1.77E+09 | 53.00% |
| RM4018+pSh-2 | 7 | 1.63E+09 | 1.03E+09 | 63.27% |
| RM4018+pSh-3 | 0 | 1.57E+09 | 1.50E+09 | 95.74% |
| RM4018+pSh-3 | 1 | 1.80E+09 | 1.23E+09 | 68.52% |
| RM4018+pSh-3 | 2 | 1.10E+10 | 6.00E+09 | 54.55% |
| RM4018+pSh-3 | 3 | 7.00E+09 | 5.67E+09 | 80.95% |
| RM4018+pSh-3 | 4 | 4.67E+09 | 4.06E+09 | 87.00% |
| RM4018+pSh-3 | 5 | 3.67E+09 | 2.97E+09 | 81.00% |
| RM4018+pSh-3 | 6 | 4.67E+09 | 3.67E+09 | 78.57% |
| RM4018+pSh-3 | 7 | 7.00E+08 | 2.33E+08 | 33.33% |
| DH5a+pSh-1 | 0 | 1.30E+07 | 1.30E+07 | 100.00% |
| DH5a+pSh-1 | 1 | 1.27E+09 | 1.27E+09 | 100.00% |
| DH5a+pSh-1 | 2 | 2.00E+09 | 1.98E+09 | 99.00% |
| DH5a+pSh-1 | 3 | 1.83E+09 | 1.84E+09 | 100.18% |
| DH5a+pSh-1 | 4 | 1.60E+09 | 1.60E+09 | 100.00% |
| DH5a+pSh-1 | 5 | 2.13E+09 | 2.09E+09 | 98.14% |
| DH5a+pSh-1 | 6 | 1.73E+09 | 1.71E+09 | 98.65% |
| DH5a+pSh-1 | 7 | 1.50E+09 | 1.49E+09 | 99.00% |
| DH5a+pSh-2 | 0 | 7.67E+06 | 7.66E+06 | 99.96% |
| DH5a+pSh-2 | 1 | 1.40E+09 | 1.39E+09 | 98.99% |
| DH5a+pSh-2 | 2 | 1.53E+09 | 1.51E+09 | 98.28% |
| DH5a+pSh-2 | 3 | 1.33E+09 | 1.34E+09 | 100.20% |
| DH5a+pSh-2 | 4 | 1.67E+09 | 1.66E+09 | 99.78% |
| DH5a+pSh-2 | 5 | 1.03E+09 | 1.01E+09 | 98.06% |
| DH5a+pSh-2 | 6 | 2.10E+09 | 2.10E+09 | 99.98% |
| DH5a+pSh-2 | 7 | 1.67E+09 | 1.65E+09 | 99.00% |
| DH5a+pSh-3 | 0 | 1.50E+07 | 1.50E+07 | 100.22% |
| DH5a+pSh-3 | 1 | 1.50E+09 | 1.49E+09 | 99.11% |
| DH5a+pSh-3 | 2 | 1.70E+09 | 1.69E+09 | 99.12% |
| DH5a+pSh-3 | 3 | 1.97E+09 | 1.95E+09 | 98.98% |
| DH5a+pSh-3 | 4 | 1.50E+09 | 1.47E+09 | 97.98% |
| DH5a+pSh-3 | 5 | 1.87E+09 | 1.84E+09 | 98.73% |
| DH5a+pSh-3 | 6 | 1.20E+09 | 1.18E+09 | 98.03% |
| DH5a+pSh-3 | 7 | 1.93E+09 | 1.92E+09 | 99.15% |

**NOTE:** In the table, RM4018 denotes the *Arcobacter butzleri* RM4018 strain, DH5α denotes *Escherichia coli* DH5α, and the pIMM24 vector is referred to as pSh. Each strain was tested in triplicate.


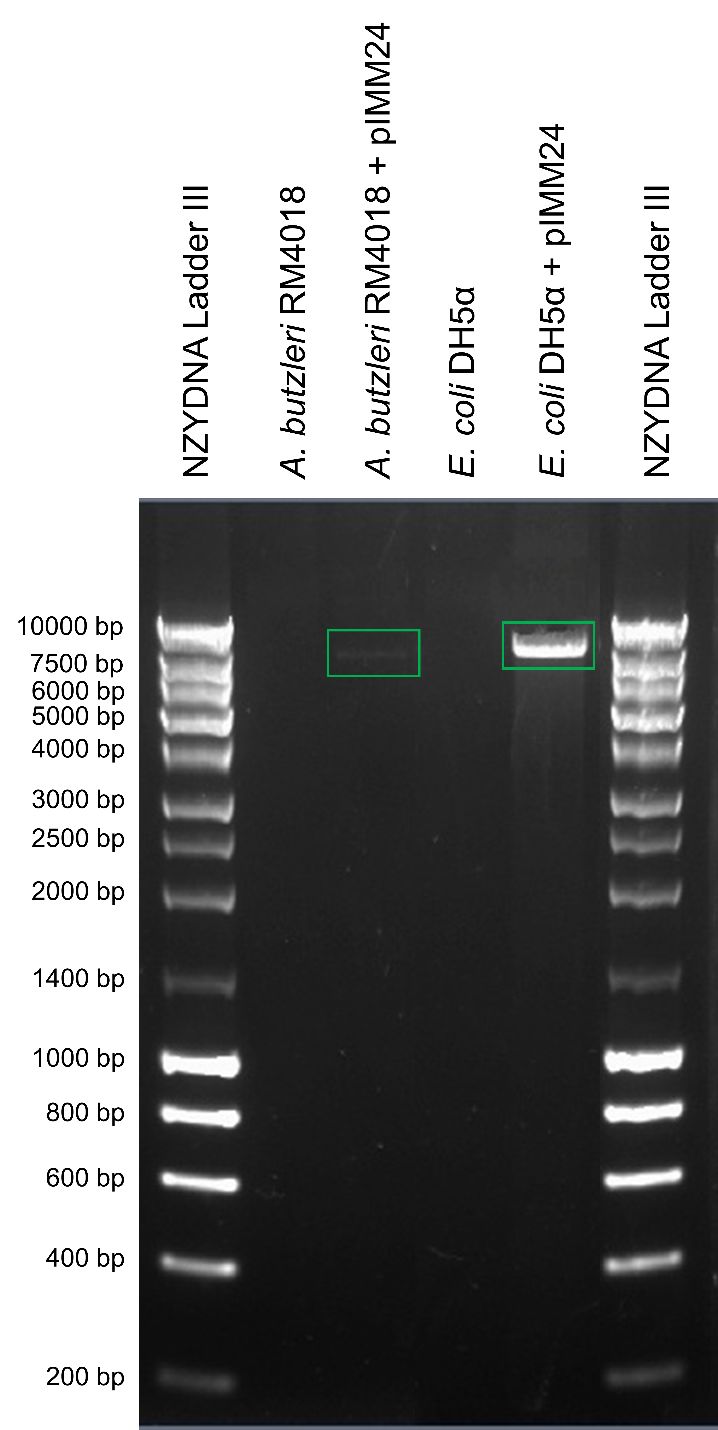


**Figure S2.** Agarose gel electrophoresis of EcoRV linearized GeneJET extracted DNA (0.8% agarose, 80 V, 105 min). DNA digestion was carried out using directly 10 µL of the GeneJET extracted plasmidic DNA with FastDigest EcoRV endonuclease (37 ºC/5 min; ThermoScientific, Vilnius, Lithuania). Plasmid DNA (7,425 bp) is indicated by green squares.

**Table S2.** Quantification of DNA from each strain using GeneJET (ThermoScientific, Vilnius, Lithuania).

|  | **DNA yield (ng/µL)** | **Abs 260/280** | **Abs 260/230** |
| --- | --- | --- | --- |
| *A. butzleri* RM4018 | -1,5 | 1,86 | 0,17 |
| *A. butzleri* RM4018+pIMM24 | 2,1 | 1,34 | -0,37 |
| *E. coli* DH5α | -1,0 | 1,87 | 0,20 |
| *E. coli* DH5α + pIMM24 | 102,3 | 1,54 | 0,76 |
